# Establishing and Scaling-Up Clinical Social Franchise Networks: Lessons Learned From Marie Stopes International and Population Services International

**DOI:** 10.9745/GHSP-D-15-00057

**Published:** 2015-06-12

**Authors:** Sarah Thurston, Nirali M Chakraborty, Brendan Hayes, Anna Mackay, Pierre Moon

**Affiliations:** ^a^​ Independent Consultant, Marie Stopes International, New York, NY, USA; ^b^​ Population Services International, Washington, DC, USA; ^c^​ Marie Stopes International, New York, NY, USA

## Abstract

In many low- and middle-income countries, a majority of people seek health care from the private sector. However, fragmentation, poor economies of scale, inadequate financing, political opposition, a bias toward curative services, and weak regulatory and quality control systems pose serious challenges for the private sector. Social franchising addresses a number of these challenges by organizing small, independent health care businesses into quality-assured networks. Global franchisors Marie Stopes International (MSI) and Population Services International (PSI) have rapidly scaled their family planning social franchising programs in recent years, jointly delivering over 10.8 million couple-years of protection (CYPs) in 2014—up 26% from 8.6 million CYPs just 1 year prior. Drawing on experience across MSI’s 17 and PSI’s 25 social franchise networks across Africa, Asia, and Latin America and the Caribbean, this article documents the organizations’ operational approaches, challenges faced, and solutions implemented. The organizations provide intensive capacity building and support for private-sector providers, including clinical training, branding, monitoring quality of franchised services, and commodity support. In addition, franchising programs engage providers and clients through behavior change communication (BCC) and demand generation activities to raise awareness and to attract clients, and they implement initiatives to ensure services are affordable for the lowest-income clients. Social franchise programs offer the private sector a collective platform to better engage government in health policy advocacy and for integrating into new public health care financing and procurement mechanisms. The future of social franchising will require developing approaches to scale-up and sustain the model cost-effectively, selectively integrating other health services into the franchise package, and being responsive to evolving health care financing approaches with the potential to contribute to universal health coverage.

## Abstract (precis)

Family planning social franchising has succeeded in countries with an active private sector serving low- and middle-income clients, with services provided mostly by mid-level providers, such as nurses and midwives. Key support for social franchising includes: clinical training and supportive supervision, help building sustainable businesses, marketing and demand creation, and mechanisms to make services affordable for clients. The forward agenda includes selectively introducing other priority health services, improving cost-effectiveness of the model, and promoting sustainability and health system integration.

## Teaser

Family planning social franchising has succeeded in countries with an active private sector serving low- and middle-income clients, with services provided mostly by mid-level providers, such as nurses and midwives. Key support for social franchising includes: clinical training and supportive supervision, help building sustainable businesses, marketing and demand creation, and mechanisms to make services affordable for clients. The forward agenda includes selectively introducing other priority health services, improving cost-effectiveness of the model, and promoting sustainability and health systems integration.

## INTRODUCTION

In many low- and middle-income countries, a majority of men and women seek health care from the private sector, be it a clinic, a pharmacy, or a roadside kiosk. As an example of this trend, analysis of data from the Demographic and Health Surveys since 2000 indicates that 51% of the population in sub-Saharan Africa, 66% in Southeast Asia, and 79% in South Asia sought health care in the private sector in response to children’s illness,^1^ without meaningful differences between the rich and poor.

Patients often prefer the private sector over public hospitals or clinics for their perceived availability, including shorter waiting times and better customer service orientation.^2^ Two systematic reviews have determined that these perceptions are well founded.^3^^,^^4^ A number of assessments of the effectiveness of private-sector strategies on health outcomes have been conducted,^5^^-^^7^ although results have been largely mixed or inconclusive.^3^^,^^4^^,^^8^

In most contexts, the private sector is highly fragmented and lacks economies of scale, adequate financing, and quality control and assurance systems. Among the strategies used to engage the private sector, social franchising has been shown to improve quality, client satisfaction, and access to services,^9^^-^^12^ especially preventive services that may be underprovided due to lower profit potential.

Social franchising aims to address challenges of oversight, quality, and scale in the private sector by organizing small, independent health care businesses into quality-assured networks. Social franchising is among the only models to leverage existing private-sector infrastructure to expand access to and improve quality of services. The model applies the principles of commercial franchising to achieve social goals. Under the social franchising model, the *franchisor* is the entity that organizes private clinics into quality-assured networks while providing a comprehensive support package, ranging from training, quality monitoring, and commodities to branding, marketing, behavior change communication (BCC), and demand generation support. Clinic members, called *franchisees*, retain ownership and management of their facilities but maintain compliance with franchise quality standards and monitoring.

Social franchising organizes small, independent health care businesses into quality-assured networks.

The franchising model explicitly addresses failures in providing high-quality, high-priority, accessible health services,^13^ and it speeds the dissemination of new health technologies in the private sector. However, to deliver its promise of health impact, social franchising must be brought to scale. Scaling the franchise model poses a number of operational challenges that Marie Stopes International (MSI) and Population Services International (PSI), two of the largest global franchisor entities, have been working through in recent years.

This article describes MSI and PSI’s franchising approaches, from launch through scale-up. It begins with background on MSI and PSI clinical social franchising programs before setting out key contextual factors in policy environments and health markets that support franchising success. The paper goes on to examine social franchising from the supply side (that is, MSI and PSI inputs for building the capacity of franchisee clinics) followed by an exploration of the support given to the demand side (for example, client engagement, BCC, and strategies for equitable access). The article closes with a discussion of the opportunities for integrating social franchising into health systems and of the future of social franchising, specifically examining how MSI and PSI work toward the long-term goals of franchise sustainability and contributing to achieving universal health coverage.

## MSI AND PSI SOCIAL FRANCHISING PROGRAMS

As international global health organizations, MSI and PSI approach franchising with the same core objective: to strengthen the private sector’s role in meeting health care needs in countries where poor health and inequities in health outcomes persist. Influenced by their respective histories and the evolution of their franchising models, the MSI and PSI approaches to social franchising share many characteristics but differ on a number as well.

Both organizations operate a *fractional* franchise model, whereby the organizations franchise a specific package of services while franchisee clinics continue to offer other services without MSI or PSI involvement. This is in contrast to well-known, full-format commercial franchising models, such as McDonald’s, in which the franchisor has extensive control over all aspects of the franchisee’s business. The fractional approach allows for rapid scale-up of services because it builds on the franchisee’s existing basic health care and business infrastructure and takes advantage of the clinic’s existing client base to expand access to services. It also provides a solid foundation for introducing preventive and other high-impact but underprovided services (Box 1).

**BOX 1. What Is the Difference Between Social Marketing and Clinical Social Franchising?**

Both MSI and PSI deliver social marketing and clinical social franchising programs; the focus of this paper is on clinical social franchising.

**Social marketing** programs make health products, including contraceptives, accessible and affordable through private-sector outlets, such as pharmacies and shops, while using commercial marketing techniques to achieve specific behavioral goals.^14^

**Clinical social franchising** focuses on building the capacity of existing private-sector health facilities (typically clinics and, in some cases, hospitals) and their staff providers to deliver important yet often underprovided health care services, such as contraceptive methods that require a clinical procedure (i.e., implants, intrauterine devices, or permanent methods).

While social marketing and clinical social franchising share a number of functional requirements, such as provider and client behavior change communication, clinical social franchising requires more-involved clinical training and monitoring, from provider recruitment and skills transfer to quality assurance.

**Figure:**
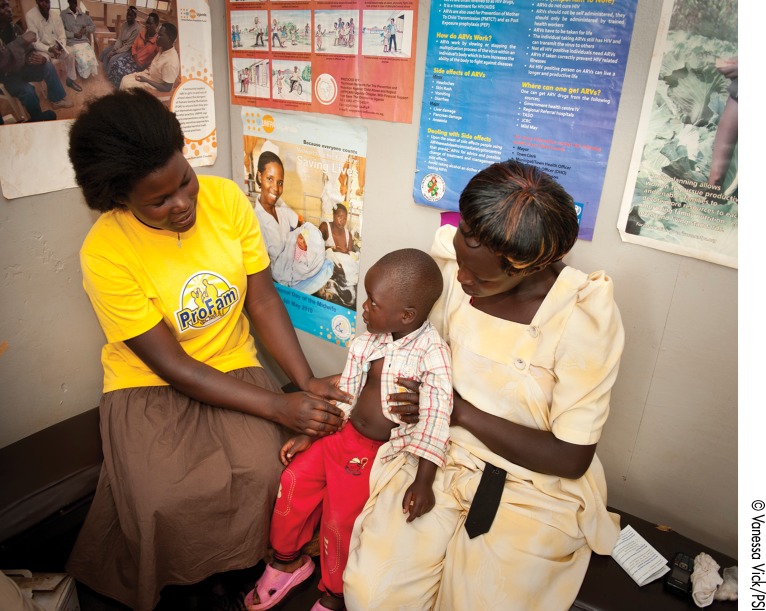
A midwife in Uganda, operating under the PSI ProFam social franchising brand, examines a young child. Most PSI franchise networks began with family planning services, but some have expanded their offerings into new health areas, such as child health.

**FIGURE 1.**
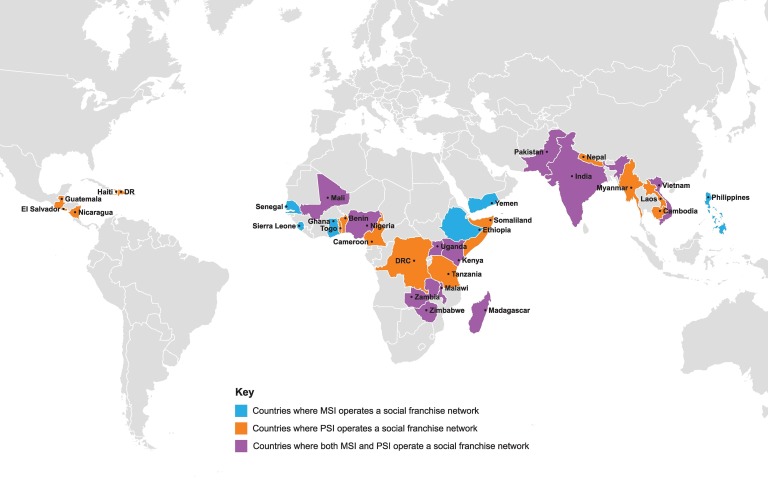
Global Footprint of MSI and PSI Social Franchise Programs in 2013 Abbreviations: DR, Dominican Republic; DRC, Democratic Republic of the Congo; MSI, Marie Stopes International; PSI, Population Services International.

MSI began franchising in 2001 in Latin America after decades of experience operating an extended network of wholly owned clinics, which in 2014 comprised more than 620 facilities in 37 countries. Social franchising provided a mechanism to accelerate access to family planning and other sexual and reproductive health (SRH) services by working with existing private providers in contexts where it was either not feasible or not cost-effective to open new MSI clinics. MSI currently operates social franchise networks in 17 countries in Africa and Asia (Figure 1).

MSI’s franchising focus on family planning has most often involved adding missing health technologies and services, especially voluntary long-acting reversible contraceptives (LARCs), to franchisee clinics’ existing service platform, alongside strengthening counseling and short-acting method provision, in order to expand contraceptive choice. As detailed in the companion paper in *Global Health: Science and Practice*, 70% of the 1.24 million family planning clients served through MSI’s franchisees in 2014 chose a voluntary LARC or permanent method.^15^

PSI’s work in social franchising began more than 20 years ago in Pakistan, emerging from its focus on product social marketing to strengthen the existing private sector. PSI identified the need to complement health products with access to clinical services, and social franchising provided a strategic expansion opportunity to better serve clients with both products and the services. Currently, PSI social franchise networks operate in 25 countries across Africa, Asia, and Latin America and the Caribbean (Figure 1).

PSI approaches franchising with a broader range of services beyond family planning. In some cases, PSI has introduced new products, for example, malaria rapid diagnostic tests, but most often the approach focuses on changing provider behaviors and practices to improve the quality of existing services. While most PSI franchise networks began with contraceptive services, PSI’s networks in southern Africa initiated franchising with HIV services and in Somaliland with child health services. PSI’s franchise networks expand their service offerings into new health areas—for example, child health or tuberculosis—as franchisee capacity develops and financing opportunities and donor interests align.

### Growth in Franchise Networks

**FIGURE 2.**
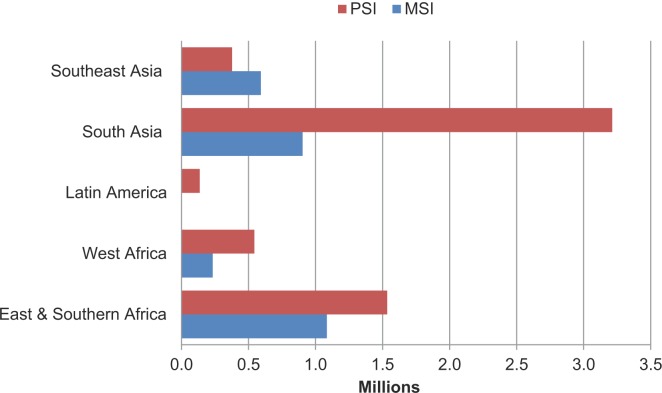
Couple-Years of Protection (CYPs) Delivered Through MSI and PSI Social Franchise Clinics in 2013 Abbreviations: MSI, Marie Stopes International; PSI, Population Services International. Data are from Viswanathan et al.^16^

Between 2008 and 2014, MSI and PSI’s social franchising footprints grew exponentially. In 2013, the organizations’ combined social franchising programs delivered 8.6 million couple-years of protection (CYPs) (Figure 2). In just 1 year, the CYPs they delivered rose by 26%, to over 10.8 million, based on preliminary 2014 data from MSI and PSI internal records. (CYPs provide a measure of the amount of time a couple will be protected against unintended pregnancy per unit of the particular contraceptive method used. One CYP is the equivalent of 1 year of protection from unintended pregnancy for 1 couple.)

MSI and PSI together delivered over 10.8 million couple-years of protection in 2014.

**FIGURE 3.**
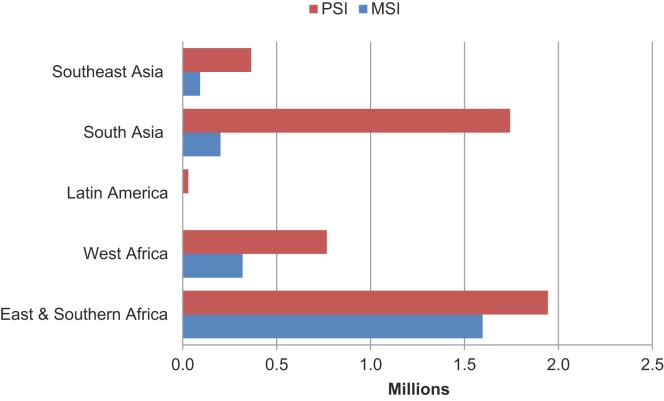
Disability-Adjusted Life Years (DALYs) Averted Through MSI and PSI Social Franchise Clinics in 2013 Abbreviations: MSI, Marie Stopes International; PSI, Population Services International.

The overall health impact of the family planning services provided by MSI and PSI social franchise networks can be measured in terms of the disability-adjusted life years (DALYs) averted. DALYs are calculated as the sum of years of life lost due to premature mortality in the population and the years lost due to disability for people living with a health condition or its consequences. One DALY can be thought of as 1 lost year of “healthy” life, and the sum of DALYs across a population can be thought of as a measurement of the gap between current health status and an ideal health situation whereby the entire population lives to an advanced age, free of disease and disability.^17^ In 2013, PSI’s social franchising family planning services averted more than 2 million DALYs in Africa, and MSI averted about 1.6 million DALYs in the continent (Figure 3). Similarly in Asia, PSI averted about 2 million DALYs while MSI averted nearly 0.5 million DALYs.

## FEASIBILITY AND POSITIONING OF FRANCHISE NETWORKS IN NEW MARKETS

MSI and PSI make the decision to launch social franchising in a new country context after a thorough assessment of the health market and policy environment. Each organization conducts feasibility assessments to determine whether the context is conducive to franchising and how a new franchise might be best positioned in the health market. Through experience, MSI and PSI have honed their approach to identifying and evaluating the contextual factors that matter most (Box 2).

**BOX 2. Where Can Social Franchises Succeed? The Factors That Matter the Most**

MSI and PSI’s experience has shown that the ideal scenario for establishing a social franchise occurs in health markets where:

- The private medical sector, especially the outpatient sector, has adequate institutional capacity
- Poor and underserved client groups currently seek care from private providers
- The public sector is overburdened and/or unable to meet unmet need for family planning
- The government is interested in and supportive of developing, regulating, or contracting the private sector
- Clients or third-party payers are willing to buy health services offered in small private-sector outlets
- Clients or third-party payers are able to pay for services, either through out-of-pocket payments, health insurance, or other demand-side financing schemes
- Adequate resources are available for franchise set-up and ongoing management
- The policy environment is favorable to task sharing whereby mid-level providers can offer the franchised package of services

### Determining Feasibility

In addition to assessing whether a particular context is conducive to franchising, feasibility assessments also offer a platform for engaging with stakeholders in government and civil society. Social franchising may be new to in-country audiences, especially in cases where previous public-private partnerships focused on tertiary care rather than small and medium enterprise.

Because fractional franchising works through the existing private sector, a base level of activity in the private sector must be present, and these providers must be actively serving catchment areas where low- and middle-income clients live or work. Franchising has flourished in countries where lower-income clients *already* seek care in the private sector, such as in India, Kenya, Pakistan, and Uganda. Conversely, the model has not flourished as well in places without a strong private sector or where the private sector serves mainly wealthier clients, for example, in Angola, Rwanda, and Sierra Leone.

MSI and PSI work through the existing private sector in areas where private providers already serve low-income clients.

Experience shows, however, that few contexts are perfect for franchising; barriers exist in any health market. Franchising success depends on early identification of barriers so that strategies to overcome them can be developed and implemented.

### Positioning a New Franchise Brand

MSI and PSI use commercial brand positioning principles to introduce new franchise brands. Brand positioning refers to the space that a brand occupies in the minds of clients in relation to alternatives. MSI and PSI work to define and communicate what is special about their franchise. What positive attributes does the brand signal to clients? How will it deliver its promise of quality, choice, and affordability?

MSI and PSI communicate these brand attributes through targeted marketing of their social franchise networks to a wide potential client base. A range of traditional marketing approaches may be used—for example, signs and billboards or radio advertisements—but the specific approaches used depend largely on what types of marketing and advertising are permitted for health services in a given country.

MSI has adopted a strategy of uniform brand identity through the *BlueStar* brand and uses this name, logo, and brand across country platforms with only a few exceptions. To better customize the brand to the local context, an appropriate tag line such as *health care network* (in the local language) is also used.

Social franchising in PSI originally grew out of the needs of each country, and so PSI enabled the brands, products, and services of each social franchise to evolve according to the marketing and health needs of each country. More recently, PSI has begun promoting a regional brand strategy, where possible, to enable coordination and economies of scale. The *Tunza* and *ProFam* brands are common in East and West Africa, respectively, the *Sun Quality Health* network operates in Asia, and *Red Segura* serves Latin America. The approach still allows for local customization—for example, branding in Swahili in East Africa and in Spanish in Latin America—but equally keeps costs down by allowing new franchises to leverage existing brand strategy, marketing materials, and other inputs.

### Shared Risk to the Brand

Brand is an important part of the value that social franchising adds to participating clinics. Positive brand association can increase client volumes and improve clinic profitability.^15^ It may also raise the prestige of franchise providers among their colleagues and in their communities, although more research is needed to test and quantify this effect.

Although the fractional franchise model that MSI and PSI operate can be more rapidly scaled than a full franchise model, fractional franchising carries inherent brand risk. Because franchisees continue to offer non-franchised services that MSI and PSI do not regulate or monitor for quality, an ever-present risk exists that the non-franchised services could be of poor quality or could result in an adverse event that could damage the franchise brand, not only in one franchisee clinic but across the network.

In recent years, both MSI and PSI have begun partnerships with Netherlands-based *SafeCare,* a certification scheme taking root in East Africa that acts as a quality assurance, improvement, and accreditation scheme for an overall clinic. The certification program aims to help providers deliver safe and quality-secured care to their patients, according to internationally recognized standards. This type of program may be one solution to address the inherent limitations of a fractional franchise model that has historically focused on specific health interventions, such as family planning or maternal health.

To date, the risks of fractional franchising have not materialized into brand damage for either MSI or PSI. Still, the tension in fractional franchising is clear, and so special attention must be paid to franchisee selection and brand management. Specific franchisee recruitment strategies differ between MSI and PSI, but both organizations tend to favor a shared set of characteristics in providers, such as being employed full-time in their private practice and demonstrating interest in providing the franchised package of services (Box 3).

**BOX 3. Who Makes for Good Franchisee Candidates?**

As an absolute minimum standard, MSI and PSI require all prospective franchisees to be licensed to practice and the clinic to be registered with the national regulatory authority.

Beyond this requirement, recruitment strategies for MSI and PSI differ but may favor providers who:

- Are employed full-time in their private practice, rather than part-time while also being employed in the public sector
- Demonstrate interest in SRH services, for example, by previously offering some SRH services (or other target areas) but not necessarily the full range
- Are supportive of SRH services, for example, supportive of family planning
- Are located in a low-income area and are willing and motivated to serve the poor
- Are not located too close to other outlets for franchised services (for MSI, this includes clinics owned and operated by MSI)
- Are female, in contexts with strong client preferences for female providers, such as in Pakistan and Yemen

## THE SUPPLY SIDE OF FRANCHISING: WORKING WITH PROVIDERS

Choosing the right providers for franchising and effectively supporting them to deliver franchised services—many of which are new to the providers—are essential franchise management functions.

It can be tempting to selectively choose only the highest-capacity providers for recruitment into the network. However, such providers do not typically operate in areas where low- and middle-income clients live. Further, working with lower-capacity providers to improve quality and expand their range of services is an important part of improving standards of care in the private sector, a key promise of franchising and one that selectively choosing only high-capacity providers negates.

In the franchisee recruitment process, MSI and PSI work to strike the right balance between acceptable standards and room for improvement. Critically, prospective franchisees must be willing and committed to improving their clinical quality and expanding their range of services to include franchised services, typically encompassing primary care and preventive services with lower profit margins than curative services.

Both MSI and PSI have had good experiences working with owner-operated facilities. When franchise providers own their facility, buy-in and accountability for agreed quality improvements can be more easily ensured. For recruitment, both organizations seek out clinics run by mid-level provider cadres, such as clinical officers, midwives, and nurses. Experience has shown that the behavior and practices of doctors is harder to influence.

Mid-level providers, such as nurses and midwives, tend to be a better fit for social franchising than doctors.

### Recruiting Providers Into the Network

Mapping existing facilities in target regions is the first step in franchisee recruitment. Using a combination of official records and site visits for scoping, MSI and PSI identify the number and distribution of prospective franchisees. This mapping work is combined with analysis of population and health data to determine the concentrations of underserved clients.

MSI and PSI visit prospective franchisees and assess their capacity using a standardized tool that is customized to the country context. Standardization and transparency in the selection process is important. It provides a basis to explain to clinics that are not selected what they can do to improve their prospects for future consideration.

Prospective franchisees need to see value in joining the franchise network. In Malawi, MSI’s affiliate Banja La Mtsogolo (BLM) engages current franchisee providers to speak with prospective franchisees in a group setting. Hearing from other providers they deem credible helps prospective franchisees understand the benefits of membership and how franchisee and franchisor can best work together.

There is no exact right number of franchisees for a network. Volume depends on program objectives, expected client volumes, and likely attrition rate. Attrition due to relocation, retirement, or drop-out happens in the course of managing a franchise network. De-franchising due to breaches in compliance is also a reality. Good recruitment practices help keep attrition and de-franchising to a minimum.

**Figure:**
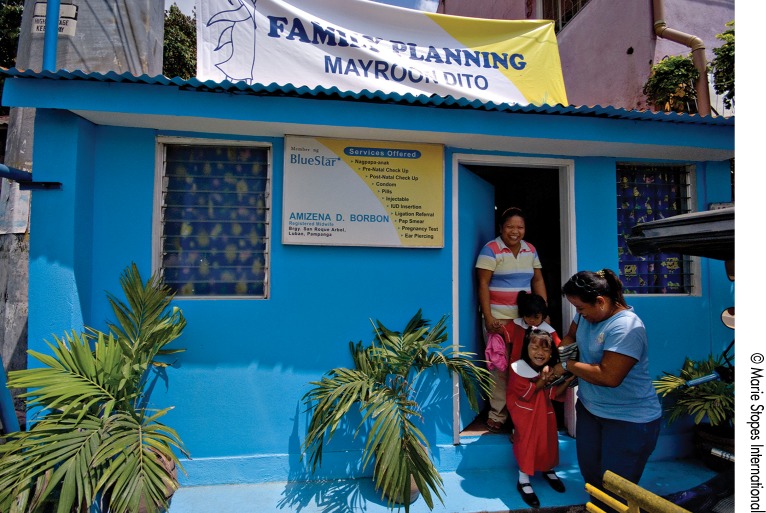
In the Philippines, a client with her children leaves a private family planning clinic run by a midwife. The clinic is part of the MSI BlueStar social franchising network, which generally recruits mid-level provider cadres, such as nurses and midwives.

### Clinical Training and Monitoring Quality of Services

Clinical training, provider certification, and ongoing quality assurance are important functions of franchise management (Box 4). Competency-based classroom and practical training relate specifically to the services being franchised but include general good clinical practices as well.

**BOX 4. How Do MSI and PSI Quality-Assure Social Franchise Providers?**

- Establish clinical minimum standards aligned with national standards
- Issue clinical governance policy, which includes requirements for clinical quality audits, client satisfaction surveys, and incident reporting protocols
- Deliver competency-based training on the package of franchised services, including training for procedures, comprehensive client counseling, infection prevention, management of clinical emergencies, and referrals
- Facilitate supply of appropriate commodities and equipment
- Provide quarterly supportive clinical supervision and routine use of quality monitoring audits (see supplementary material)
- Conduct independent audits of the quality assurance system and practices (for PSI)

For example, MSI and PSI train their franchisees on a full range of family planning information and methods including comprehensive client counseling, provision of voluntary LARCs, refresher training on short-acting methods, and referral for voluntary permanent methods. Building franchisees’ skills to offer voluntary LARCs involves classroom and practical training modules covering quality monitoring; counseling; infection prevention; method insertion and removal; and preparedness to handle medical emergencies. Once franchisees demonstrate competence in all clinical quality areas, they are branded as part of the franchise network and can begin delivering franchised services.

Both MSI and PSI undertake a regular program of monitoring and support for their franchisees. Through this supportive supervision, the franchise managers work to reinforce clinical skills; ensure uninterrupted access to medical commodities; and help franchisees build demand for franchised services in their communities. MSI and PSI standards of practice require at minimum quarterly monitoring visits for each franchisee; however, many franchisees require more frequent visits, especially in the early months when skills are first being developed. (See supplementary material for sample indicators used by MSI to assess franchisees’ clinical standards during quality audits.)

Supportive supervision visits offer the opportunity to hear from providers on a range of issues and to identify potential problems, including problems stemming from low provider confidence. In Uganda, MSI’s *BlueStar* providers were trained to offer a full range of short- and long-acting contraceptive methods. However, during a client tracing survey, a significant number of respondents reported that their franchisee provider had not counseled them about intrauterine devices (IUDs). After following-up with franchisees, Marie Stopes Uganda learned that many franchisees, although trained to provide IUDs, felt less confident with counseling and inserting IUDs than they did with providing implants and short-acting methods. *BlueStar* retrained franchisee providers on IUD counseling, insertion, and removal, and it established a mentoring program between more-experienced franchisees and those reporting low confidence. As a result, IUD provision among *BlueStar* franchisees in Uganda grew from 12% of LARCs provided in 2012 to 24% at the end of 2013 (data from internal MSI sources).

Strong communication and effective interaction with providers is key. As a backdrop to all interactions with franchisee providers, PSI field staff use medical detailing or provider BCC techniques and skills. Medical detailing and provider BCC are similar processes (as they relate to franchising) that involve a PSI staff member visiting individual health clinic providers or owners of pharmacies or drug shops to educate them about a particular product or service and to promote that product or service. During medical detailing, PSI staff will explain how a particular product works, its benefits, and its side effects. This information is intended to influence provider/owner behavior, and the provider can also transfer the information to clients during counseling and service provision.

PSI has found that training providers on clinical skills does not always translate into immediate uptake or practice of those skills in the clinical setting (for a variety of reasons). PSI’s provider BCC approach addresses the training-to-practice gap by complementing training with follow-up medical detailing but also by managing the overall relationship with the provider effectively and maximizing opportunities to support and influence provider behaviors and provider-client interaction skills. The specialized communications skills of the medical detailers, modeled after those used in the pharmaceutical industry, allow PSI field staff to identify underlying provider needs, motivations, and/or barriers and biases to performing the desired behavior or service. PSI field staff can then offer the provider a variety of relevant and actionable solutions designed to meet the individual provider’s needs and motivations—solutions that are valuable to the provider. The medical detailers distribute resources such as literature, counseling charts, and patient brochures to assist providers in adopting the desired behaviors.

### Helping Franchisees Build Sustainable Businesses

The quality improvements that clinics achieve through their franchise membership continue to deliver impact so long as the franchisee remains in business. Helping franchisees strengthen and expand their businesses is often a key part of the value promised by the franchisors. For example, business management training for franchisees was developed by Banyan Global and has been used by PSI and MSI to train franchisees in a number of countries, including Ethiopia, Malawi, Sierra Leone, and Zimbabwe. The training, covering such topics as assessing one’s business, recordkeeping, financial statements and analysis, cash flow plans, and accessing finance, is popular with franchisees. Some franchise networks approach business strengthening by linking franchisees to services present in the market: PSI is currently piloting various approaches, including recruiting specialist “business advisors” in Kenya and outsourcing business support to franchisees to a third-party supplier in Tanzania. In addition, several networks have used their platform to connect franchisees to private and public health insurance companies in a bid to help diversify sources of revenue for franchised businesses.

Access to affordable capital for business expansion is limited in many developing countries, especially for small health care businesses with little loan collateral. MSI, PSI, and Society for Family Health (SFH) have worked to align franchising with programs designed to help small and medium enterprises develop business plans and secure capital. Franchisees in Ghana, Kenya, and Nigeria are eligible for small-business loans facilitated by the Medical Credit Fund (MCF) of PharmAccess. Small loans, in the amounts ranging from US$2,000 to $5,000, can be used to finance clinic renovations, procure new medical equipment, improve technology, buy assets, or upgrade administrative systems. Once franchisees pay off their first loan, they are eligible for follow-on lending.

PSI is also partnering with the private sector to scale-up social franchising while improving financial sustainability. For example, in rural Uganda PSI is collaborating with a corporate partner to support the PSI franchise and ensure greater access to maternal health facilities and commodities for pregnant women. Alongside this partnership, PSI’s social franchising programming in Uganda is developing a sustainable supply chain for maternal health products through wholesale pharmacies and drug shops.

### Taking Provider-Side Approaches to Scale

Social franchising can make a great difference in quality and access to care at the community level.^19^ However, to achieve a wider and sustained health impact, social franchising must operate at scale. Scaling a model that requires a high level of interpersonal interaction and support is challenging and costly; therefore, MSI and PSI must work to develop quality-assurance mechanisms that are replicable and cost-effective at scale.

Technology has a role to play in reaching that scale cost-effectively. In Zimbabwe, MSI’s *BlueStar* network has created a mentoring program that uses WhatsApp, a free mobile phone application that allows real-time communication and problem solving between new franchisees and more-established clinic mentors. PSI has begun using technology for improved data collection and better, more-targeted monitoring and supportive supervision visits. In Nepal, for example, PSI franchise staff are equipped with tablet computers that they use to collect real-time data, but the tablets are also programmed with a variety of clinical reference guides and behavior change tools. For staff stationed in hard-to-reach areas, the tablets allow them to keep up-to-date on new and revised materials efficiently, as well as to improve timely data collection.

Technology can help scale social franchising mechanisms cost-effectively.

Strategies such as improved use of technology help overcome operational challenges to make franchising more cost-effective at scale. Finding such cost-effective solutions is an important precursor to positioning franchise networks for longer-term sustainability.

## ENGAGING CLIENTS TO OVERCOME DEMAND-SIDE BARRIERS

Social franchising is often understood to be a supply-side intervention aimed at improving provider capacity. While intensive work with providers plays a big part in implementation, engaging clients is also key. Franchisees themselves are at the frontline of community-level marketing and client engagement in their catchment areas, as fractional franchising builds off their brand equity.

However, MSI and PSI play an important support role, namely to target key underserved groups with health information and ensure communities are aware that quality, affordable services are available at their local franchisee clinic. Franchisors use BCC approaches to encourage positive health-seeking behavior and demand generation strategies to raise awareness about franchised services and the health benefits they offer. BCC and demand generation messages may be delivered through various channels including mass media and interpersonal engagement. In addition, both MSI and PSI selectively use financing mechanisms, such as vouchers, to reduce barriers to access and to attract clients to the franchised clinics.

### Attracting Clients With BCC and Demand Generation

BCC and demand generation activities undertaken by MSI and PSI work to reduce information and behavioral barriers that can restrict service uptake. One key BCC and demand generation strategy is interpersonal engagement. Reaching clients where they live and work is both highly interactive and time-intensive. However, this work is an important part of the franchise network’s added value; small owner-operated facilities rarely have the time or resources to undertake person-to-person engagement at scale.

Franchisors use behavior change communication to generate demand for franchised services.

PSI’s Latin America franchise, *Red Segura*, works inside garment factories, visiting the factory floors to educate young female employees about their SRH choices and how and where to access services through franchisees. In India, PSI’s network conducts door-to-door mapping and household visits, working to reach young married women in their homes. In Pakistan, MSI’s *Suraj* network takes a similar approach by engaging female health educators to conduct door-to-door visits with information on SRH topics and franchisee locations.

### Making Services More Affordable Through Demand-Side Financing

Considerable evidence suggests that out-of-pocket payments reduce use of preventive health services in low- and middle-income countries.^20^ To expand access for low-income clients, MSI and PSI have piloted and adopted different mechanisms to reduce client out-of-pocket payments. These include setting price caps, encouraging sliding-scale fees, distributing targeted vouchers, and offering periodic free service days, often coupled with provider training.

While many of these mechanisms have demonstrated success, they are not without challenge. For example, price caps can be difficult to enforce in practice, and differential, sliding-scale fees introduce the risk of friction between clients or between client and provider. Vouchers and free service days have increased service uptake in several MSI and PSI franchise networks, with some evidence of improved equity in service access.^21^^,^^22^ Voucher programs, however, can be costly to run and therefore should be used in appropriate circumstances as an equity intervention and not solely as a marketing device.

Voucher programs, although often costly to run, may improve service access for the poor.

In Pakistan, PSI integrated safe motherhood vouchers into its client engagement approach. The safe motherhood voucher provided clients with subsidized access to 8 services, including 3 antenatal care (ANC) visits, normal delivery, referral for cesarean section if needed, postpartum family planning, and postnatal care visits. In the pilot study, women who purchased vouchers had a significantly higher uptake of ANC and institutional delivery than women who did not purchase vouchers.^21^ When the intervention was expanded, results indicated that while poorer women were less likely to use ANC and institutional delivery, those living in areas with vouchers had a significantly increased likelihood of doing so than those living in control areas.^22^

In Madagascar, MSI has successfully used vouchers to increase access to family planning for underserved groups. From 2012 to 2014, more than 50,000 poor women were able to access family planning using a voucher. Research in 2013 showed that 85% of voucher clients were poor, as measured by multidimensional poverty index (MPI), compared with 25% of franchisee family planning clients overall. Moreover, franchisee client records showed that the volume of paying clients rose significantly between 2012 and 2014, indicating that well-targeted vouchers can be used to attract poor or underserved clients without compromising the paying-client revenue model that franchisees often rely on (data from MSI internal sources). In 2014, MSI combined a voucher program for adolescents with training on youth-friendly services for franchisees. Where franchisees previously served very few adolescents, more than 3,000 adolescents a month are now using the vouchers to access voluntary family planning services from franchisees (data from MSI internal sources).

## HEALTH SYSTEMS INTEGRATION

Social franchise networks offer a platform to more successfully engage the private sector in countries’ wider health systems. Two important areas gaining traction are articulation of a private-sector voice in health policy advocacy and integration of the private sector into new public health care financing and procurement mechanisms.

### Engaging the Voice of the Private Sector in the Health System

Fragmentation in the market results in missed opportunities for the private sector to engage in dialogue on important national health care issues. Through social franchise networks, private providers can engage with a collective voice, amplifying their agenda and offering a platform for government and other stakeholders to effectively engage with them. In some countries, groups of franchisors have formed franchise associations, providing a unified platform for knowledge sharing and advocacy. For example, in Kenya, the Association of Social Franchising for Health brings together more than 1,000 private providers from 6 franchise networks, with the aim of entering into policy engagement and dialogue with the government and other decision makers.

Advocacy for task sharing and task shifting provides examples of successful use of this collective voice for policy change on issues directly affecting lower- to mid-level provider cadres. In Mali, PSI’s *ProFam* network is changing norms around public-sector task shifting by piloting an approach to task shift IUD provision to midwives in private *ProFam* clinics. After advocating the change in national health guidelines, PSI conducted training of trainers for Ministry of Health staff to ensure the ministry would be able to adopt and replicate the task shifting approach independently. In 2009, only 4 public-sector locations provided IUD services; by 2013, trained midwives were providing IUDs in 248 community health centers.

Task-sharing policies, however, do not always change in a direction that is favorable to franchising, as PSI’s *Sun Quality Health* franchise experienced. In Laos and Myanmar, the *Sun Quality Health* franchise trained nurse-owned private facilities to deliver voluntary LARCs. Training was complete and the franchise up and running when policies changed in both countries. In Myanmar, the government reversed permission for nurses to offer IUDs and implants; in Laos, private-sector provision of LARCs was banned. Through its franchise network, PSI immediately engaged partners in both countries and, in Myanmar, has successfully advocated policy change back to allow nurses to provide voluntary LARCs.

Additional important advocacy areas include private-sector licensing requirements. In Ethiopia, MSI’s *BlueStar* network was able to successfully advocate a 12-month delay and reconsideration of minimum facility size standards for clinic licenses, regulations that would have negatively impacted the ability of smaller-sized clinics to offer a wide range of services to their clients.

### Linking the Private Sector to Opportunities in the Broader Health System

In many countries, governments see the private sector as an important player in the health system and, in response, are prioritizing better regulation of the private sector. Further, many governments are working to integrate a better-regulated, higher-capacity private sector into structures and mechanisms previously reserved for the public sector. Examples of this integration include permitting private health facilities to access public medical commodity supplies and linking private providers with emerging health financing schemes such as national health insurance.

Reliable access to medical commodities is central to high-quality service delivery. Across many countries, the Ministry of Health or other government entity runs commodity procurement and distribution programs. However, these programs are often closed to the private sector or very difficult for small, independent private providers to access due to bureaucratic barriers, high minimum order quantities, or poor information about eligibility criteria. On behalf of their franchise networks, MSI and PSI have tapped into government supply chains in a number of countries. In some instances, commodities are accessible free of charge, while in others subsidies or purchasing pool discounts are available. These cost savings can then be passed on to franchisee clients in the form of lower service fees.

A second and strategically important area where inroads are being made is integrating the private sector into health financing schemes run by the government. Although national health insurance schemes are not yet established in most countries where MSI and PSI work, the movement is expanding in keeping with the principles of universal health coverage.^23^^,^^24^ In countries with a more developed insurance sector, both MSI and PSI are helping their franchisees gain insurance accreditation, which translates into reduced out-of-pocket payments for clients. In the Philippines, MSI’s *BlueStar* network has worked successfully with 188 of its 267 franchisees to pass the stringent accreditation process by the national health insurance, PhilHealth. Even after passing, claims submission challenges persist for small franchisees. Insurance claims must be submitted on paper at locations some distance from franchisees’ towns and villages, requiring them to close their clinics to make the journey. In response, *BlueStar* began integrating claims submission into its routine supportive supervision for franchisees. Now, *BlueStar* staff collect and submit quarterly national insurance claims, allowing franchisees to focus on client care.^25^ Further, in an effort to help bridge cash flow constraints caused by weeks or months of waiting for claims to be paid by PhilHealth, *BlueStar* offers short-term loans to help franchisees cover costs while waiting for their reimbursements.

## LOOKING FORWARD: THE FUTURE OF SOCIAL FRANCHISING

For social franchising to be effective, both in terms of health impact and financial sustainability, franchisors such as MSI and PSI and their stakeholders should consider the following priority areas, many of which are already underway: developing and using uniform metrics to measure success; addressing operational challenges and improving cost-effectiveness; integrating new franchised services; adapting to new health financing landscapes; and continuing to play an active role in health systems strengthening.

### Toward Development and Use of Uniform Metrics

Social franchising has shown promise in its ability to improve quality and increase uptake of underutilized preventive and primary care services among low- and middle-income clients. However, franchise results are often not comparable. MSI and PSI are active participants in the Social Franchising for Health community of practice (see www.sf4health.org) to define and use standard metrics, and both organizations are strong advocates of using research to quantify the social franchising model’s potential to achieve equity, cost-effectiveness, and health market expansion.

### Opportunities for Domestic Autonomy

MSI and PSI continue to work through operational questions that have implications for financial sustainability and for the sustainability of health impact delivered through franchise networks. Both organizations are interested in increasing scale to improve access to quality services. However, it is not clear if the current quality assurance systems, including supportive supervision, provider behavior change, and clinical monitoring, are scalable in a cost-effective way. Without ongoing donor subsidy, it is also unclear whether networks structured around preventive health services such as family planning can deliver value for franchisees as small businesses. Finally, the goal of franchisors’ financial sustainability can be debated, as we do not know what will happen to service availability, quality, and equity if franchisors leave the market. As noted below, the emergence of increased domestic finance for health (for example, social health insurance schemes) as a replacement for donor subsidy may provide some opportunities, provided that such schemes are accessible to the non-state/private sector (including small private providers) and that they adequately reimburse for preventive care measures such as family planning.

Experiences from successful social marketing programs including Social Marketing Company (SMC) Bangladesh and GreenStar Pakistan suggest that country-level programs can move toward autonomy from previous international franchisor support structures. However, this autonomy is at a governance level, and donor support often remains important for commodities and operations. Clinical social franchise networks may also have opportunities for greater or full domestic autonomy, as seen with the Well Family network in the Philippines^25^ (not affiliated with MSI or PSI), and the need for donor subsidy is likely to decline in countries where domestic financing systems are able to cover health care costs for the poor while a growing middle class may increase the number of clients willing and able to pay for services.

Ultimately, franchise network sustainability will depend on franchisors creating value for their franchisees and for the health systems they are supporting.

### Opportunities for Service Integration

Today, family planning services comprise the majority of services delivered through social franchise networks.^16^ That being said, most PSI franchise networks include integrated provision of HIV counseling and testing, management of childhood illness, and fever case management including malaria treatment. PSI sees this scope expansion as a cost-effective way to leverage existing provider relationships, take advantage of improvements in cross-cutting clinical areas such as infection prevention, and deliver a franchise offering that covers a larger share of the revenue-generating services offered by their providers.

Integrating other priority health services beyond family planning into the social franchise package can be a cost-effective way to leverage existing provider relationships.

MSI’s approach largely continues to focus on the reproductive health needs of women but is testing an expanded basket of services in several countries to meet those needs beyond family planning. Offering a broader package of services can also facilitate access to family planning, whether for young people interested in a broader range of SRH services or for women with young children seeking maternal and child health services. For example, both MSI and PSI are increasing access to postpartum contraception by strengthening franchisee family planning counseling during ANC visits and by introducing postpartum IUD services to franchisees already providing obstetrics services.

Irrespective of the degree of service integration, both of these approaches have historically encountered challenges with vertical funding streams of priority health areas. Increasingly, however, donors are funding an expanded package of franchised services through the same financing mechanism.

### Health Financing and Health Systems Strengthening

With momentum building around universal health coverage, there is increasing attention on public financing of health care for the poor and underserved and on a reduction in cash payments at the point of service. The benefits achieved to date of organizing small private providers into networks could translate into reduced transaction costs for public payment mechanisms as well as ensure that the small private providers that many of the poor currently use can be included in the universal health coverage agenda.

Social franchising can support the universal health coverage agenda by potentially reducing public payment transaction costs.

While franchisors need to continue to be advocates for including their network members into payment schemes (small private providers in the case of most of the networks), other stakeholders should also remember that the private sector is a vast resource and that an organized, quality-assured private sector can be more easily accredited for inclusion in emerging insurance networks, similar to MSI’s successful integration into the Philippines’ National Health Insurance Programme (NHIP).

While the discussion continues on where social franchising will be in 10 or 15 years, it is clear that organizations such as PSI and MSI are working in diverse, dynamic, and quickly evolving health markets. In many countries, there will continue to be a compelling case for franchise networks to scale health care access with the benefit of subsidies. However, franchisors will also need to be responsive to changing health care needs and to the evolution of how health care is paid for. In some countries, such changes may mean a business model that works toward health impact goals while optimizing services in a predominantly cash-based private health care market. This could evolve into autonomous domestic franchise networks or a process of graduating individual high-capacity franchisees out of franchise programs. In other countries, evolution may mean integration into public financing systems.

## CONCLUSION

The benefits of organizing and quality-assuring small health care businesses that serve the poor, at scale, presents tantalizing opportunity in the context of the universal health coverage debate. With a growing body of evidence on social franchising and operational experience across a greater number of country contexts and health areas, the coming years promise to be an exciting time for this model and for the access, quality, and equity objectives that the global health community shares.

## Notes

**Peer Reviewed**
